# Investigating the Food and Drug Administration Biotherapeutics Review and Approval Process: Narrative Review

**DOI:** 10.2196/14563

**Published:** 2021-03-04

**Authors:** Samuel Bonet Olivencia, Farzan Sasangohar

**Affiliations:** 1 Department of Industrial and Systems Engineering Texas A&M University College Station, TX United States; 2 Center for Outcomes Research Houston Methodist Hospital Houston, TX United States

**Keywords:** biotherapeutics, drug approval, drug review process, model-based systems engineering

## Abstract

**Background:**

The development, review, and approval process of therapeutic biological products in the United States presents two primary challenges: time and cost. Advancing a biotherapeutic from concept to market may take an average of 12 years, with costs exceeding US $1 billion, and the product may still fail the US Food and Drug Administration (FDA) approval process. Despite the FDA’s practices to expedite the approval of new therapies, seeking FDA approval remains a long, costly, and risky process.

**Objective:**

The objective of this paper is to explore the factors and gaps related to the FDA review and approval process that contribute to process inefficiencies and complexities as well as proposed methods and solutions to address such gaps. This paper also aims to investigate the available modeling efforts for the FDA approval process of therapeutic biological products.

**Methods:**

A narrative review of literature was conducted to understand the scope of published knowledge about challenges, opportunities, and specific methods to address the factors and gaps related to the review and approval of new drugs, including therapeutic biological products. Relevant peer-reviewed journal articles, conference proceedings, book chapters, official reports from public policy professional centers, and official reports and guidelines from the FDA were reviewed.

**Results:**

Of the 23 articles identified in this narrative literature review, none modeled the current FDA review and approval process structure to address issues related to the robustness, reliability, and efficiency of its operations from an external point of view. Although several studies summarize the FDA approval process with clarity, in addition to bringing to light the problems and challenges faced by the regulatory agency, only a few attempts have been made to provide solutions for the problems and challenges identified. In addition, although several reform models have been discussed, these models lack the application of scientific methodologies and modeling techniques in understanding FDA as a complex sociotechnical system. Furthermore, tools and methods to assess the efficacy of the models before implementation are largely absent.

**Conclusions:**

The findings suggest the efficacy of model-based systems engineering approaches for identifying opportunities for significant improvements to the FDA review and approval process. Using this holistic approach will serve several investigative purposes: identify influential sources of variability that cause major delays, including individual, team, and organizational decision making; identify the human-system bottlenecks; identify areas of opportunity for design-driven improvements; study the effect of induced changes in the system; and assess the robustness of the structure of the FDA approval process in terms of enforcement and information symmetry.

## Introduction

### Background

The introduction of new medicines and treatments into the market is a time- and cost-consuming process that is closely supervised and regulated to ensure the safety and effectiveness of the products. This process takes, on average, 12 years, and the estimated average cost of taking a new drug from concept to market exceeds US $1 billion [1]. After significant expenditure of the manufacturer’s time and resources, many drugs fail to achieve approval late in the process.

Regulatory agencies, such as the European Medicines Agency (EMA) and Health Canada, are responsible for promoting and protecting public health in their respective geographic areas through the evaluation and supervision of medicines for humans before their release into the market. In the United States, these regulatory functions fall under the responsibilities of the US Food and Drug Administration (FDA), which is the oldest comprehensive consumer protection agency in the US Federal Government [2]. Factors such as expanded federal regulations, increasing complexity of drugs and devices, and the growth of the pharmaceutical industry have expanded the role of the FDA, which is now one of the largest consumer safety agencies in the world [1].

The increasingly complex regulatory environment and expenses associated with drug development have been criticized for the resultant lag in the release of new pharmaceuticals into the drug market. In addition, the FDA risk aversion approach has forced companies to go overseas and has encouraged medical tourism [3]. A subset of advocacy groups and experts in drug regulation and policies are demanding more rapid development, approval, and release of new products because they consider the current process to be risk averse, slow, and inefficient [4]. In the past, numerous major safety incidents occurred because of drugs released into the market with little or no regulation from the FDA. As a result, weakening or removing FDA regulations for pharmaceuticals is not an option [1]. However, the FDA has created programs to facilitate the development and expedite the approval of drugs that treat serious conditions or fill an unmet medical need [5]. These drugs receive a fast-track designation, which makes them eligible for (1) more frequent meetings and written communication with the FDA; (2) accelerated approval or priority review; and (3) rolling review, meaning that the review application can be submitted and reviewed in sections rather than as a complete application. In addition, the US Congress issued the Prescription Drug User Fee Act (PDUFA) to authorize the FDA to collect fees from applicant companies to invest in resources to accelerate FDA review and approval operations [6]. Despite these efforts, FDA scrutiny remains a long, costly, and risky process, making it challenging for patients to have timely access to potentially useful medications and treatments [7].

As with any new drug, the FDA’s long scrutiny affects the review and approval of new biopharmaceutical products, which have become an important sector of the pharmaceutical industry in the United States, the country with the largest market for biopharmaceuticals (around 33% of the global market) [8]. Reports from the National Science Foundation reveal that the US biopharmaceutical sector accounts for the largest single share of all US investments in research and development (R&D) [8]. This fast-growing sector is in a critical position in which therapeutic biological products represent over a third of all new drugs in clinical trials or are awaiting approval from the US FDA [8].

In the past, there has been a clear difference between biotechnology industries and pharmaceutical industries; however, this has become increasingly blurred as many pharmaceutical industries are increasing their presence in biopharma. These large companies have the capital to invest in high-risk research, development, review, and licensing of new therapeutic biological products. However, there is a relevant sector of start-up biotechnology companies that are more vulnerable to the high risks and uncertainties in product development. The biopharmaceutical development process is complex, and small companies must invest significant amounts of capital and human resources. They assume risks in terms of execution, safety, efficacy, and license approval during the regulatory process. The initial financial conditions play a significant role in the financial and strategic planning of the start-up companies. Many of these start-up companies finance their R&D projects by partnering with big pharmaceutical companies or other bioscience corporate partners, securing capital from angel investors, or obtaining government or small business grants [9]. Large pharmaceutical companies, which have the capabilities, operational scale, and capital, create corporate alliances with small biotech companies with the understanding that the larger pharmaceutical entity will have a considerable amount of control and profits over the start-up. Statistics show that there is a 20% likelihood for a bioproduct to progress from the initiation of phase 1 clinical trials all the way to market approval [9]. When partnering with a start-up, companies, sponsors, and investors assume a large portion of the risk and uncertainty associated with the development of the therapeutic biological products. Although there is a risk associated with the upfront investment in R&D and clinical trials, estimated to be in tens of millions of US dollars, there is also a considerable amount of risk regarding the time and cost associated with navigating the FDA approval and licensing process for a biopharmaceutical product [9]. In addition to the associated time and cost complexities, there are other challenges, some unique and others in common with any other new drug, that characterize the release of a therapeutic biological product into the market.

### Objectives

The goal of this paper is to explore the factors and gaps related to the FDA drug review and approval process that contribute to process inefficiencies and complexities as well as proposed methods and solutions to address such gaps. The focus of this paper is to understand the constraints and challenges in the drug review and approval process identified by researchers who investigated FDA operations and to identify the models that have been applied to understand, evaluate, analyze, and suggest improvements to the FDA drug review and approval process.

## Methods

A narrative review of the literature was conducted to understand the scope of the published peer-reviewed knowledge about challenges, opportunities, and specific methods to address these factors and gaps related to the review and approval of new drugs, including therapeutic biological products.

### Search Strategy

First, official reports and guidelines from the FDA official website were retrieved to document the biotherapeutics review and approval process. The authors familiarized themselves with the FDA review and approval process for new drugs, including therapeutic biological products, and identified relevant terms for use in the search of relevant peer-reviewed journal articles, conference proceedings, book chapters, and official sources from public policy professional centers. Several databases including Science Direct, ABI/Inform Complete-ProQuest, MEDLINE (PubMed), and Google Scholar were searched using the terms “FDA” combined with “regulation” AND “model” AND “approval” to identify sources related to the FDA review and approval process. In addition, the term “FDA” was combined with “systems engineering,” “change management,” and “quality by design” for the initial search, to identify sources proposing models and alternative approaches to improve and reform the FDA review and approval process.

### Study Selection

The first search yielded 11,296 sources. These sources were screened, and a total of 9400 sources were excluded based on the following exclusion criteria: (1) inaccessibility to full text, (2) not peer-reviewed (exceptions were made for articles coming from public policy professional centers), and (3) duplications. The remaining 1896 sources were assessed for eligibility based on the following inclusion criteria: (1) published between 2000 and 2017; (2) included cost and time considerations; (3) included content about the FDA review and approval process for medical devices, drugs, and biotherapeutics, which are complex products with a longer and generally more costly pathway for approval in comparison with other FDA regulatory processes (medical devices were not excluded from the search to identify work in that area that could be applicable to the review and approval process of drugs and biotherapeutics) [10]; and (4) included content about the operationalization of the FDA review and approval process. On the basis of these criteria, 41 sources were selected for full-text review. The full-text review of the remaining 41 sources was conducted to include sources that were most relevant to the scope of this study: (1) understand the constraints and challenges in the drug review and approval process identified by researchers who investigated FDA operations and (2) identify the models that have been applied to understand, evaluate, analyze, and suggest improvements to the FDA drug review and approval process. After the full-text review, 23 sources, including peer-reviewed articles, conference proceedings, book chapters, and articles from public policy professional centers, were included in the narrative review (Table 1).

**Table 1:** Sources included in the narrative review.

Sources (reference)	Title
Conko and Madden [7]	Administrative Law and Regulation
Das and Almonor [11]	A Concurrent Engineering Approach for the Development of Medical Devices
Alexander and Clarkson [12]	A Validation Model for the Medical Devices Industry
Tsai and Erickson [9]	Early-Stage Biotech Companies: Strategies for Survival and Growth
Rathore and Winkle [13]	Quality by Design for Biopharmaceuticals
Gernaey and Gani [14]	A Model-Based Systems Approach to Pharmaceutical Product-Process Design and Analysis
Kourti and Davis [15]	The Business Benefits of Quality by Design (QbD)
Medina et al [16]	Design for FDA: A Predictive Model for the FDA’s Decision Time for Medical Devices
Medina et al [17]	Supporting Medical Device Development: A Standard Product Design Process Model
Baylor [18]	Regulatory Approval and Compliances for Biotechnology Products
Conner et al [19]	The Biomanufacturing of Biotechnology Products
Lawrence et al [20]	Understanding Pharmaceutical Quality by Design
Aksu et al [21]	QbD Implementation in Biotechnological Product Development Studies
Briggeman et al [22]	The Proper Role of the FDA for the 21st Century
Kinch [23]	2015 in Review: FDA Approval of New Drugs
Thierer and Wilt [24]	The Need for FDA Reform: Four Models
Van Norman [1]	Drugs, Devices, and the FDA: Part 1: An Overview of Approval Processes for Drugs
Van Norman [10]	Drugs and Devices: Comparison of European and US Approval Processes
Williams [4]	Food and Drug Administration Drug Approval Process: A History and Overview
Williams et al [3]	Health Options Foreclosed: How the FDA Denies Americans the Benefits of Medical Research
Djuris and Djuric [25]	Modeling in the Quality by Design Environment: Regulatory Requirements and Recommendations for Design Space and Control Strategy Appointment
Horner et al [26]	Process Modeling in the Biopharmaceutical Industry
Joshi et al [27]	Optimization of Ion Exchange Sigmoidal Gradients Using Hybrid Models: Implementation of Quality by Design in Analytical Method Development

## Results

### Review and Approval of New Drugs

The overall timeline for the release of a new drug into the market can be divided into 2 main phases: (1) R&D and (2) review, approval, and licensing. The first stage of the R&D phase, which includes preclinical trials and 3 phases of clinical trial, involves an exchange of information between the applicant and the FDA review board. Both parties are in communication, and after each stage, the applicant must provide updated information to the FDA. Even when this flow of updated information is established, the license approval of a new drug application may take a long time because of the prolonged FDA application review process. According to the rules and procedures of the FDA, the review process of a new drug application cannot start unless the application is fully submitted. An exception is made for those new drugs accepted under a rolling review status, where the application can be submitted and reviewed in sections.

The review of a new drug application consists of 6 major steps that include reviewing clinical trial results, planning and execution of the product label, and manufacturing site inspections. In the specific case of therapeutic biological products, the current FDA review and licensing are regulated following the guidelines of *The Program*, a review program created by the reauthorization of the PDUFA Act in 2018 (PDUFA VI), which is valid for the duration of the current version (until the year 2022). *The Program* was created with the intention of increasing the communication and transparency between the FDA review team and the applicant, to increase the efficiency and effectiveness of the first review cycle. In addition, *The Program* decreases the number of review cycles necessary to approve a biologics license agreement (BLA), which is a highly complex application [28]. These revisions also provide additional review clock time for the agency to meet with the applicant during review as well as to address review activities that occur late in the review cycle [28]. According to the timeline established in the Center for Drug Evaluation and Research (CDER) 21st Century Review Process Desk Reference Guide, the total estimated time for a standard review of a BLA under *The Program* is 12 months, and it is 8 months for a priority review [29]. All these efforts to expedite and streamline the process come with a cost that is pushed to the applicant. PDUFA VI authorizes the FDA to collect fees from companies that submit BLAs. For example, an application including clinical trials may have a fee of approximately US $2.5 million dollars [6].

In addition to the time and money constraints in the process, the review and approval of a drug involve multiple FDA resources and constant communication with the applicant through phone calls, emails, and meetings. The personnel assigned to review an application vary according to the type of submission and product. In general, an application is reviewed by a team of professionals from different disciplines. The review team has to deal with the flow of not only new submissions but also resubmissions. A company can resubmit an application to answer all the deficiencies indicated by the FDA in the initial review [30]. The resubmissions may put a strain on FDA’s normal operations by sharing resources between both types of submissions.

Other constraints and challenges have been identified by researchers who investigated FDA’s operations [1,4,10,18,19,23]. Van Norman [1] points out the complexity of the approval process and emphasizes that the main challenges for pharmaceuticals are in terms of cost and time. Similarly, Williams [4] emphasizes the criticism and controversy that the FDA has confronted because of the lengthy approval process and that the FDA has been accused of conflict of interest because of the user fees collected from sponsors and drug manufacturers to support the drug approval process. In addition, the author discusses critics’ claims that FDA’s operations are slower and less efficient than that of the EMA in the European Union (EU), even when there is little evidence available to support the criticism [4]. Van Norman [10] compared the European and US approval processes with the purpose of presenting their similarities and differences as well as the perceived challenges faced by each. His work emphasizes how both approval processes are similar, except that the US approval process is completely centralized, whereas the EU has 4 possible pathways for drug approval: (1) centralized through the EMA (mandatory for some classes of drugs, such as those used in the treatment of oncological diseases and diabetes, among others), (2) national (each EU state has its own procedure), (3) by mutual recognition (drugs approved in one EU state can obtain marketing authorization in another EU state), and (4) decentralized (manufacturers can simultaneously apply for authorization in more than one EU state). In addition, Van Norman [10] presents data that weaken the claim that the FDA is significantly slower than the EMA.

Another relevant challenge faced by the FDA is the lack of transparency in nonpublished drug trial data. According to Van Norman [10], this issue results in challenges associated with the production of systematic reviews and meta-analyses that are essential to public health and safety. Promoting information symmetry (information equally accessible to all parts involved, including regulators, industry representatives, and consumers) must be a central function of the FDA. This issue suggests the need for an assessment of the current review and approval structure. In addition, it is necessary to investigate methods to support individual, team, and organizational decision making to balance the process structure in terms of enforcement and information.

In a similar manner, pharmaceutical quality oversight has been a major issue that the FDA has addressed over the years, establishing strict regulatory standards to ensure the safety and efficacy of the products. Modeling to comply with quality regulation standards has been another research area of interest in the academic community. Specifically, importance has been given to formulation and process modeling, addressing the quality by design (QbD) concept, first introduced in 2004 as part of the Pharmaceutical cGMPs (Current Good Manufacturing Practices) for the 21st Century Initiative [13,25,27]. QbD assists both the industry and the FDA in implementing a scientific approach by targeting the desired product quality throughout the design and development process. This is different from the traditional quality by testing methodology as it encourages the definition of a design space early in the process development. The design space works as an acceptable operating range for the critical process parameters. Changes in the process within the design space are acceptable by the FDA. Any movement outside the design space is considered to be a change that must be approved by the FDA. Consequently, a regulatory postapproval process must be initiated by the manufacturer and submitted to the FDA for review and approval [13].

The QbD approach was implemented in other FDA review programs before being adopted by the Office of Biotechnology Products because of the complexity of its application in the development and manufacturing process of biotechnology products. Since the implementation of QbD principles for pharmaceutical development, multiple benefits have been identified, but certain challenges have been raised, for example, (1) determining common terminology between the industry and the FDA [20], (2) the provision of training programs to industry representatives [21], (3) lack of understanding and trust among all stakeholders involved [21], and (4) the associated costs of implementing QbD in product development and regulatory processes [21]. One of the major challenges in the implementation of QbD has been to manage the surveillance of legacy products approved before the implementation of the QbD principles [13]. The integration of QbD into the drug development process adds a new complete level of interconnections and communications between the industry and the FDA. A common understanding of QbD and the steps involved is necessary to facilitate communication between both parties [15,20]. Therefore, modeling and architecting the FDA network flow structure will require addressing the inter- and intraconnections in the drug application review process. In addition, such modeling approaches must provide a clear representation that leads to a common understanding of the QbD approach, highlighting the elements relevant to the entity in charge of the risk-based drug development process (industry) as well as the entity responsible for reviewing and monitoring the drug application submissions (FDA).

### Modeling the FDA Regulatory Process

#### Models and Points of View

Most of the reviewed academic literature that modeled FDA regulatory processes presents efforts in modeling from the applicant’s point of view. The purpose of modeling from this view is to enhance the chances of compliance with the regulatory requirements and recommendations. Most work in this area applies to the development of medical devices, with a special focus on design [11,12,16,17]. For example, Medina et al [17] developed a standard product design process model to support medical device development. Even when the work includes FDA regulations that complement the medical device development process, the focus is directed toward modeling the process to comply with FDA regulations rather than addressing the efficiency and robustness of the FDA regulatory process. A relevant aspect of their work is the application of model-based systems engineering (MBSE) tools to model the medical device design and development process. Medina et al [16] applied unified modeling language to model the relationships among the different elements (classes) in the development process. This research was later extended to the development of a predictive tool to estimate the FDA decision time for medical device approval, with the purpose of estimating the product’s time to market [16]. The relevance of applying an MBSE approach to the FDA approval process is because of its suitability for analyzing complex systems as networks of interrelated elements that include people, facilities, policies, laws, regulations, internal and external institutions, and technologies, among other elements. In a system as complex as the FDA drug approval process, the application of an MBSE approach may provide the following benefits: (1) facilitate communication among the various stakeholders involved; (2) provide a set of models (abstractions to manage size and complexity) that serve as a tool to analyze the effect of changes to the system; (3) allow compare and contrast analysis of the *as-is* and *to-be* solutions; and (4) allow the exploration of multiple system alternatives concurrently with minimal risk, among other benefits [31].

A review of the available literature reveals a lack of research on the approval process from the regulatory agency’s point of view. In addition, only a few studies have applied modeling approaches with direct application to pharmaceutical products and process design and analysis [13,27]. Most research efforts were industry-driven and directed toward the incorporation of modeling tools into drug development and production practices, with the purpose of enhancing the process by reducing the cost and time to market of the products [14,26].

#### Reform Models and Change Management

In a stochastic system typical of the FDA, regulation procedures can undergo changes at any time. The pressure that the FDA is receiving to relax the scrutiny process and expedite the time to market of critical pharmaceuticals increases the urgency to enhance and reform the regulatory agency approval process. Following this line of thinking, Thierer and Wilt [24] presented a summary of 4 models to reform the FDA approval process and change the way medical products are brought to the market. These 4 models are flexible approaches presented under the premise that the implementation of a comprehensive reform would benefit both the innovators and the patients.

The first model, developed by Williams et al [3], targets the approval of medical devices by suggesting the provision of regulatory authority to multiple private parties that will compete with the FDA on the price, quality, and timeliness of approvals. This model not only creates a competition for trust between the FDA and the private regulatory bodies but also requires the FDA to fulfill a new role of establishing quality standards and good manufacturing practices that must be monitored by private regulatory parties. As established by the authors, this reform model can also be applied to the approval of drugs [24]. This idea was first presented in 2000 by Henry I Miller [32], who proposed the creation of nongovernmental drug-certifying bodies, changing the role of the FDA from being the *certifier of products* to being the *certifier of the certifiers* [33]. An implementation of a model such as this will create a significant change in the drug approval process as new stakeholders will be integrated into the review process, whereas the FDA’s responsibility would be limited to a higher-level review. If analyzed as a network, this will require a new set of interrelations and modified information flow.

The second reform model was discussed by Klein and Tabarrok [33], the objective of which was to eliminate the FDA monopoly on drug approval by allowing manufacturers to market their products in the United States once they have gained approval in other major markets, such as the EU [24,33]. The term *international reciprocity* is used to refer to this approach. Similar to the first approach, this model requires the FDA to compete with other international regulatory agencies for the business of drug manufacturers.

The third model presented is more in line with the perspective of the current critics of FDA practices. The model proposes not having to wait until further clinical trials to release a drug for use when safety and efficacy have been demonstrated in the initial clinical trials. A proposal which can be referred to as *free-to-choose medicine*, developed by Conko and Madden [7], suggests a dual-track system for patients and doctors consisting of (1) deciding to stick with the current FDA procedures or instead (2) selecting a *free-to-choose track* option that provides freedom to the patient, with the advice of their doctors, to make an informed choice of using an experimental drug for which safety and efficacy have been demonstrated but FDA approval has not yet been obtained [7,24]. The implementation of this dual-track system requires a commitment from the FDA and the manufacturing companies to promote information symmetry. This reform is similar to what the Independent Institute calls *the sensible alternative*, which supports voluntary certification. This alternative proposes to keep the FDA as a voluntary institution where companies submit their application because of a belief in the integrity and cooperation of the agency, not because it is mandatory. Drugs approved under this alternative must be labeled as *not FDA approved* [33].

The fourth model, established by Briggeman et al [22], was developed based on the idea that the FDA has exceeded its authority by not only assuring safety and efficacy but also making judgments about the benefits and risks of the drugs. According to the authors, that responsibility lies with doctors and patients based on their experiences with the drug [22]. The reform model suggests that the FDA must readopt the regulation model they followed in the 80s and 90s. In this model, the regulatory agency is at the top of the funnel, setting standards to measure a drug’s effectiveness based on the pharmacologic activity on the disease. The responsibility of determining the utility of the drug is deferred to the doctors, who must make decisions regarding the adoption of a drug based on real-world experiences, the characteristics of each patient, and additional clinical trials sponsored by clinics and biopharmaceutical industries [22].

The implementation of any of these 4 reform models may imply a shift in the current activities and responsibilities of the FDA and, therefore, may change the organizational structure of the regulatory agency. The addition of new internal or external regulatory sources as well as the elimination of current regulatory bodies within the FDA will have a variety of consequences in the agency network, which must be addressed and measured for effectiveness. Current FDA publications and reports on change management were created to provide guidance to industry applicants on how to manage changes in their processes and still comply with the quality regulations [20,25]. To deal specifically with organizational changes, the FDA has developed a set of manuals of policies and procedures for each of its offices. For example, the Manual of Policies and Procedures for the CDER provides policies and procedures for submitting, evaluating, coordinating, reviewing, and approving organizational changes, including the addition or elimination of organizational components [30]. However, to the best of our knowledge, no FDA publications or academic research has presented efforts to develop a flexible system-based model that shows the interconnections and information flow among the main elements of the approval process. A contribution in this area will provide the FDA with a diagnostic tool to efficiently address the change management affairs.

## Discussion

### Principal Findings

The FDA has made efforts to improve their internal drug review and approval operations through the creation of expedited review and approval tracks and the establishment of special review programs such as *The Program*, created under the PDUFA. Although these efforts have reduced the time to market of drugs, the drug approval process remains a costly and slow process. These challenges have a major impact on small-to-medium biopharma start-ups that do not have the initial capital investment required and must resort to alternate financial agreements that are not necessarily competitive for them. More importantly, it remains challenging for chronically ill patients to have timely access to novel alternative medications and treatments that could save their lives.

Suggesting changes to the review and approval of therapeutic biological products is a challenging task because of the complexity of the process. Each therapeutic biological product has different properties, meaning that each FDA review and approval process is different. In addition, interrelationships and interdependencies exist among the different stages of the process, and external and internal factors may act as influential sources of variability, causing major delays. Steps in the process may serve as bottlenecks, halting the review process. In addition, application errors and lack of required documentation on the part of the applicant can cause major delays in the review process.

To the best of our knowledge, none of the sources identified in this narrative literature review have modeled the current FDA review and approval process structure to address issues related to the robustness, reliability, and efficiency of its operations. Although several studies summarize the FDA approval process with clarity, in addition to bringing to light the problems and challenges faced by the regulatory agency, only a few attempts have been made to provide solutions for the problems and challenges identified. In addition, although several reform models have been discussed, these models lack the application of scientific methodologies and modeling techniques to understand FDA as a complex sociotechnical system. Furthermore, tools and methods to assess the efficacy of the models before implementation are largely absent. The implementation of any of these models would not only impact the FDA’s authority but may also imply a change in the current structure of the regulatory agency. Changes proposed to the approval process must be accompanied by a dynamic model of the FDA regulatory structure under the new changes suggested.

Findings from this narrative review suggest an opportunity to employ MBSE approaches to provide a systems-oriented descriptive model of the FDA approval process for therapeutic biological products as a service network. Using this holistic approach will serve several investigative purposes: (1) identify influential sources of variability that cause major delays, including individual, team, and organizational decision making; (2) identify the human-system bottlenecks; (3) identify areas of opportunity for design-driven improvements; (4) study the effect of induced changes in the system; and (5) assess the robustness of the structure of the FDA approval process in terms of enforcement and information symmetry. However, adopting this approach may pose several challenges owing to the complexity of investigating internal FDA processes externally. Researchers external to the FDA have access to the official FDA guidelines such as the CDER 21st Century Review Process Desk Reference Guide, which provides a summarized overview of the drug approval process [29]. However, as the approval process of each drug may be different because of certain characteristics of the products and the manufacturing processes, modeling the approval process network based only on those guidelines would not reflect the intrinsic variability in the FDA operations. Consequently, any generated model may have poor external validity. Although this can be seen as a limitation, it also represents an opportunity for future collaboration between the FDA and the academia. The integration of external research and internal FDA efforts could facilitate the development of novel techniques and methodologies with practical applications that will benefit the regulator, industry, and patient population.

### Limitations

Although this narrative review provides a broad, critical, and objective analysis of the current knowledge regarding the constraints and challenges in the FDA drug review and approval process and the approaches suggested in the literature to reform the FDA review and approval process, the search process and the number of sources included are not as comprehensive as it would be in a systematic literature review. The authors of this paper have expertise in the area of human and health care systems engineering; therefore, the review is limited to analyzing the problem from a systems engineering perspective, shedding light on how systems engineering methods and approaches can be applied to obtain a better understanding of the FDA review and approval process, and to verifying, validating, and complementing the proposals of experts from other areas such as public policy.

### Conclusions

The FDA review and approval of new drugs, including biotherapeutics, is a long and complex process. The process requires the involvement of multiple stakeholders internal and external to the FDA, in addition to the complexity of the interrelationships and interdependencies that exist among the different stages of the process. Literature in this area has identified challenges in the process related to cost, financial considerations, resource constraints, need for balance in terms of enforcement and information transparency, and the need for clarity in the FDA review and approval process and the requirements of the applicants. Although the FDA has implemented efforts to expedite the review and approval process, safety- and efficacy-related concerns about relaxing the FDA regulation over new drugs remain. Reform models and approaches have been proposed by experts in the area; however, these proposals lack the application of scientific methodologies and modeling techniques in understanding FDA as a complex sociotechnical system to obtain an unbiased assessment of the models’ efficacy before implementation. MBSE approaches have been successfully used to model the FDA regulatory process for medical devices from the applicant’s point of view. There is an area of opportunity to use MBSE approaches to model the review and approval process of new drugs from the regulatory agency’s point of view to address issues related to the robustness, reliability, and efficiency of its operations.

## Abbreviations

BLA: biologics license agreement
CDER: Center for Drug Evaluation and Research
EMA: European Medicines Agency
EU: European Union
FDA: Food and Drug Administration
MBSE: model-based systems engineering
PDUFA: Prescription Drug User Fee Act
QbD: quality by design
R&D: research and development
